# Revolutionizing HIV-1 Viral Load Monitoring in India: The Potential of Dried Blood Spot Analysis for Expanding Access and Improving Care

**DOI:** 10.3390/healthcare12040413

**Published:** 2024-02-06

**Authors:** Madhuri Chandane Tak, Anuradha Vaidyanathan, Anupam Mukherjee

**Affiliations:** Division of Virology, ICMR-National AIDS Research Institute, Pune 411026, MH, India; mchandane.nari@gov.in (M.C.T.); vaidyanathan.a@gov.in (A.V.)

**Keywords:** HIV/AIDS, HIV-1, viral load, dried blood spot (DBS), diagnosis, resource-limited settings, point-of-care testing (POCT), public health

## Abstract

India continues to grapple with a significant burden of HIV infections. Despite notable progress in prevention and treatment efforts, multiple challenges, such as high-risk populations, inadequate testing facilities, and limited access to healthcare in remote areas, persist. Though the Government of India offers HIV-1 plasma viral load testing at various medical centers, aiding treatment decisions and monitoring antiretroviral therapy effectiveness, enhancing care for individuals living with HIV under the National AIDS Control Program (NACP), the nation’s large population and diverse demographics further complicate its outreach and response. Hence, strategic interventions and alternative methods of testing remain crucial to curbing HIV transmission and improving the quality of life for those affected. Dried blood spot (DBS) sampling has emerged as a convenient and cost-effective alternative for HIV-1 viral load testing, revolutionizing the landscape of diagnostic and monitoring strategies for HIV infection. Though the plasma-based viral load remains the gold standard for monitoring HIV-1, DBS-based HIV-1 viral load testing holds immense promise for improving access to care, particularly in resource-limited settings where traditional plasma-based methods may be logistically challenging. DBS entails the collection of a small volume of blood onto filter paper, followed by drying and storage. This approach offers numerous advantages, including simplified sample collection, transportation, and storage, reducing the need for cold-chain logistics. Recent studies have demonstrated the feasibility and accuracy of DBS-based HIV-1 viral load testing, revealing a strong correlation between DBS and plasma measurements. Its implementation can enhance the early detection of treatment failure, guide therapeutic decisions, and ultimately contribute to better clinical outcomes for HIV-infected individuals. Hence, this review explores the principles, advancements, feasibility, and implications of DBS-based HIV-1 viral load testing.

## 1. Introduction

The present scenario of HIV infections in India is characterized by a mix of progress and challenges. Over the years, concerted efforts have led to a decline in the overall prevalence of HIV. However, India still has one of the largest populations of people living with HIV globally [1,2]. Prevention programs have contributed to decreased transmission rates, but certain groups, such as men who have sex with men, sex workers, and injecting drug users, remain disproportionately affected [3]. Stigma and discrimination continue to hinder testing, treatment, and support services, particularly in rural areas. Access to antiretroviral therapy has expanded, improving the quality of life for many individuals. Yet, gaps in viral load monitoring coverage and antiretroviral therapy (ART) persist, and ensuring sustained adherence to treatment remains crucial to preventing drug resistance.

HIV-1 viral load monitoring is pivotal in assessing treatment efficacy and disease progression in terms of HIV and AIDS management. Accessible testing facilities play a crucial role in ensuring regular monitoring for individuals on antiretroviral therapy, treatment effectiveness, adherence, early detection of treatment failure, prevention of transmission, and a personalized treatment approach. Regular monitoring of viral load levels aids healthcare providers in making informed decisions to optimize patient care and improve overall health outcomes for individuals living with HIV and AIDS. According to the latest report from the NIH, only approximately 77% of people living with HIV (PLHIV) worldwide have access to viral load testing facilities. Furthermore, the report highlights an increase in HIV-1 drug resistance in low and middle-income countries due to poor drug adherence and limited access to viral load testing [1,2]. Resistance to antiretroviral therapy in HIV-1 evolves over time, affecting various drug classes, including the latest generation of inhibitors [4]. The transmission and persistence of drug-resistant mutations have been observed, with these mutations becoming entrenched in the population [5,6]. Furthermore, emerging drug resistance to newer compounds, such as integrase inhibitors, has been documented in anti-retroviral treatment [4]. The increasing prevalence of the evolutionary persistence of drug resistance poses a significant challenge in high viral load cases, emphasizing the critical need for regular viral load monitoring [5,6,7]. Due to this, outreach programs are vital to extending testing and monitoring to underserved areas, reducing barriers like distance and stigma. Integrating these elements fosters effective HIV management, prevents complications, and promotes overall health among affected individuals. As per the UNAIDS 2023 report, the funding shortfall for HIV prevention and management programs is on the decline. There is a growing imperative to utilize these funds more intelligently and cost-effectively to ensure that essential services can reach all those who require them. Eliminating societal and structural disparities in access to HIV-related services, resources, and tools is a crucial foundation for effectively delivering accessible HIV prevention and treatment services. This approach is pivotal in safeguarding the health and well-being of individuals and forms the cornerstone of a successful HIV response [8].

**Figure 1:**
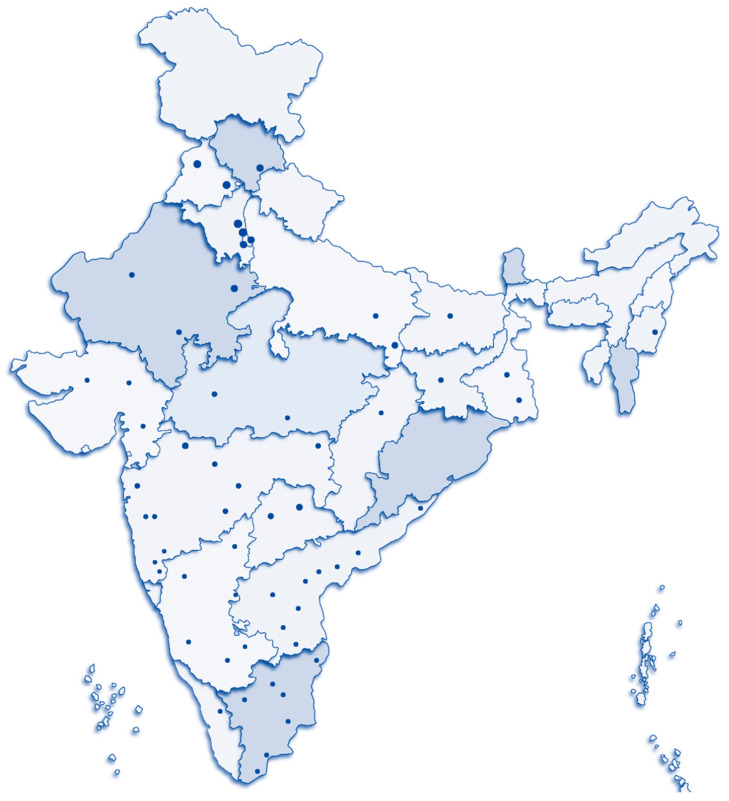
Distribution of viral load testing laboratories in India under the national program: This figure illustrates the current landscape of viral load testing laboratories in India as part of the National AIDS control program. There are a total of 64 public-sector laboratories dedicated to conducting HIV-1 plasma viral load testing across the country.

To meet the global targets of ending HIV in India, the National AIDS Control Organization (NACO) has come up with policies to reduce annual new infections and mortality by 80% by 2025–2026 through promoting universal access to quality services like viral load testing facilities to people in need of ART [3]. According to the NACO, MoHFW, the Government of India estimates, India is the third largest burdened country, with 24.1 Lakh people living with HIV and 620 anti-retroviral treatment centers and 1264 linked centers under NACP policy. These centers cater antiretroviral therapy to 1,494,000 PLHIV across the country. As per the latest NACO reports, there is a need for enhancement in ART coverage and viral load expansion in the country [9,10]. Currently, there are 64 public-sector viral testing laboratories operating under the National AIDS Control Program (Figure 1). Recognizing the importance of proficiency testing as a fundamental component of a comprehensive laboratory quality assurance program, the Indian Council of Medical Research-National AIDS Research Institute (ICMR-NARI) in Pune has been designated as the Apex laboratory responsible for conducting the nationwide HIV-1 viral load (VL) proficiency testing (PT) program. This initiative aims to enhance viral testing facilities across the country.

In this mini-review, we emphasize the critical need to diversify viral load testing methodologies, such as incorporating dried blood spots, to bridge existing gaps in expanding viral load testing across the country. Strategically implementing a spectrum of available viral load testing facilities is pivotal for extending viral load monitoring to challenging geographical locations and populations, including injecting drug users (IDUs), pregnant women, and individuals constrained by various social factors. It is imperative to recognize that each mode of testing, such as plasma samples, DBS samples, and point-of-care viral load tests, offers unique advantages and should be utilized whenever appropriate. Extensive research supports the applicability of DBS, especially in scenarios where distance from testing centers and limited collection or transportation resources hinder traditional viral load testing [11,12,13]. Implementing DBS as a sample type for viral load testing within the Indian healthcare landscape could significantly contribute to the viral load expansion program. Additionally, this review underlines the limitations of plasma and DBS testing while proposing strategies to fill the gaps, ultimately enhancing the scope of viral load testing across the country. The WHO interim technical update on implementing HIV viral load testing has succinctly summarized performance characteristics for commercially available molecular HIV viral load assays using DBS [14]. It recommends DBS specimens for viral load testing in situations where logistical, infrastructural, or operational barriers impede the use of plasma specimens.

## 2. Conventional Method of HIV-1 Viral Load Testing

**Figure 2:**
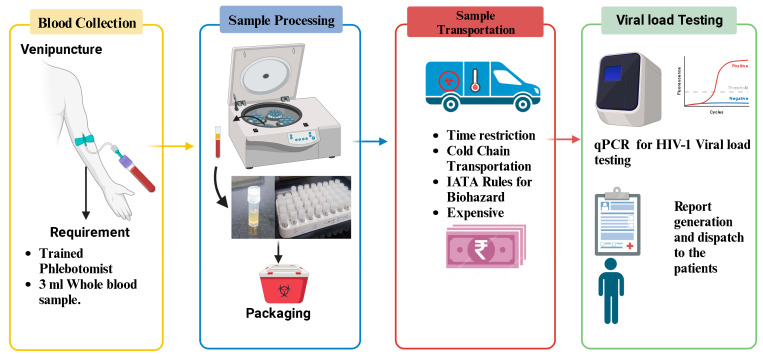
Flowchart illustrating the process of HIV-1 plasma viral load testing: This figure provides a detailed flowchart outlining the sequential steps involved in the conventional method of plasma HIV-1 viral load testing.

The conventional method of HIV-1 viral load testing primarily relies on quantifying the amount of HIV-1 RNA in a patient’s plasma, providing a crucial indicator of the viral replication activity within the body (Figure 2). This process involves the collection of a venous blood sample from the patient. The collected blood is then transported under controlled conditions to a diagnostic laboratory, where specialized equipment, such as real-time polymerase chain reaction (PCR) machines, is utilized. PCR amplifies the viral RNA in the sample, enabling precise quantification. The process requires technical expertise and an established infrastructure of laboratories. Moreover, cold-chain logistics are crucial to maintaining the integrity of the plasma samples during transportation, adding to the complexity and cost of the conventional method. Despite being the established gold standard, the conventional approach has limitations, especially in resource-limited settings, due to its demanding infrastructure requirements, cost, and dependence on a well-developed healthcare system.

## 3. The Challenges Faced in Plasma HIV-1 Viral Load Monitoring

In India, the gold standard method for monitoring HIV-1 viral load is through plasma viral load testing, which necessitates the collection and transportation of at least 3 ml of whole blood specimens to designated viral load testing facilities while maintaining a cold chain. The high-risk population in the country is primarily concentrated in geographically remote regions, such as the northeastern states of Nagaland, Mizoram, and Manipur. Additionally, other states and union territories with adult HIV prevalence rates higher than the national average include Meghalaya, Andhra Pradesh, Telangana, Delhi, Karnataka, Maharashtra, Goa, Puducherry, Punjab, Tamil Nadu, and Dadra-Nagar Haveli. Due to resource constraints in these areas, people living with HIV have limited access to viral load testing centers. Consequently, remote and hard-to-reach locations are underserved in terms of testing facilities. This situation poses challenges for PLHIV patients who may find it difficult to visit testing centers or face increased expenses and time constraints when transporting samples to distant viral load testing facilities [3,15]. Due to diverse geographical and social conditions in India, providing access to viral load testing facilities for specific population groups such as children, pregnant women, and other vulnerable individuals poses significant challenges. The prevalence of mother-to-child transmission of HIV-1 in India is notably high, with 8.78% of children born to infected mothers being affected [16]. To address this, NACO has recommended guidelines for monitoring HIV in pregnant women, emphasizing the importance of maintaining viral load suppression. This includes conducting viral load testing within 2–4 weeks of pregnancy onset and once after each trimester if the viral load remains stable. The objective of drug therapy during pregnancy is to initiate and sustain maximum viral load suppression. However, according to the UNAIDS 2021 data, India only achieved 50% viral load testing coverage among individuals with HIV on treatment in 2020, falling short of the UNAIDS-required level [17,18].

Monitoring HIV-1 viral load in resource-limited settings presents a multitude of challenges. These settings often deal with a shortage of well-equipped laboratories and adequately trained personnel for both sample collection and HIV-1 viral load testing. This deficiency in infrastructure and expertise makes it particularly arduous to establish and sustain testing facilities, especially in remote or rural areas. When testing facilities are located at a distance, the transportation of samples from these remote regions becomes problematic on multiple fronts. Not only is there the concern of maintaining sample integrity, but the process also incurs significant expenses, chiefly due to the necessity of preserving a cold chain. Proper storage conditions, including the stringent requirements of cold-chain maintenance, are imperative to safeguard sample integrity and ensure the accuracy of viral load measurements. However, the insufficiency of transportation and storage facilities can jeopardize the quality of samples and, consequently, impact the reliability of test results. As a result, the associated costs of sample transportation and cold-chain maintenance in hard-to-reach geographic regions emerge as a formidable barrier to the expansion of viral load monitoring programs.

The current situation highlights a disparity in viral load coverage for various segments of the population, including vulnerable groups such as women, children, sex workers, and other individuals living with HIV who face limited access to testing facilities in the country. Consequently, there is a pressing need to explore cost-effective alternative sample options for HIV-1 viral load testing. This is essential given the substantial financial burden associated with conducting viral load testing using plasma specimens. The goal is to reduce costs and broaden access to viral load testing for all individuals who require antiretroviral therapy [19].

## 4. Available Alternate Methods to Plasma HIV-1 Viral Load Testing

As technology continues to advance, various viral load testing methods are emerging as viable options, including point-of-care facilities such as the cartridge-based nucleic acid amplification test (CBNAAT), plasma separation cards (PSCs), and dried blood spots. These methods are becoming increasingly significant due to their user-friendly nature and cost-effectiveness in viral load monitoring. There is an opportunity to incorporate these alternate testing approaches into the viral load expansion program. Each of these resources comes with its own unique set of advantages and implementation capabilities for the viral load expansion program. However, their practical suitability in real-world settings needs to be thoroughly tested and evaluated in the field.

Table 1 clearly highlights the necessity of making alternative sample selections when dealing with resource constraints, keeping in mind factors such as time, budget, and feasibility. In such circumstances, it is worth considering the use of alternative samples. Among these alternatives, DBS stands out as a well-researched and successfully implemented method for viral load testing in various parts of the world. The ICMR-National AIDS Research Institute remains committed to enhancing patient care and services through its various national programs. In this review, we aim to provide a concise overview of the applicability of DBS specimens within our country for the viral load expansion program.

## 5. The Potential of Dried Blood Spot Analysis as a Convenient and Cost-Effective Alternative for HIV-1 Viral Load Measurement

Historically, the concept of dried blood spots as a sample type originated around the end of World War II. The DBS technique primarily employs filter paper to absorb blood samples, which are subsequently utilized for laboratory testing [20]. Robert Guthrie notably pioneered the application of DBS for phenylketonuria screening in 1960. Today, DBS finds wide-ranging utility in serological diagnosis, encompassing diseases like AIDS, hepatitis, trypanosomiasis, hepatic amebiasis, congenital rubella, and more [21].

In the early 2000s, there was a growing interest in employing DBS for therapeutic monitoring of HIV infection, particularly in resource-limited settings where access to nearby laboratories remains a substantial challenge due to constraints in human and financial resources. DBS emerges as a cost-effective alternative, notably in remote, challenging-to-reach rural areas where plasma specimen preparation and transport are hindered by costly cold-chain requirements and a scarcity of trained personnel for venipuncture and plasma separation. In light of this, recent WHO guidelines for antiretroviral therapy advocate for DBS as a viable option to enhance access to viral load monitoring for HIV, HBV, and HCV diagnosis [22].

Numerous studies have consistently demonstrated a robust correlation between DBS viral load assessments and plasma-based viral load testing [23,24]. These investigations have underscored the exceptional sensitivity and specificity of DBS specimens for detecting HIV-1 viral loads. DBS analysis presents compelling advantages as an accessible and cost-effective alternative for quantifying HIV-1 viral loads. It eliminates the need for intrusive venipuncture, streamlining sample collection and enabling self-sampling, especially in remote or resource-constrained regions where healthcare accessibility and transportation pose challenges. The dried form of DBS samples ensures convenient storage, transport, and reduced biohazard risks compared to liquid samples. Furthermore, it reduces the demand for complex cold-chain logistics, resulting in substantial cost savings and widened testing availability.

DBS analysis also demands smaller blood volumes, mitigating patient discomfort and facilitating pediatric testing. This approach proves particularly beneficial in regions where traditional plasma-based techniques face hurdles due to infrastructure limitations. DBS samples can be efficiently collected, shipped, and analyzed, promoting timely diagnosis, treatment monitoring, and therapy adjustments. These advantages firmly establish DBS analysis as a transformative tool, addressing gaps in HIV-1 viral load monitoring and advancing global HIV control and healthcare objectives.

## 6. Advantages of DBS over Plasma Specimen

Dried blood spots offer distinct advantages over plasma specimens, primarily in terms of their simplicity and cost-effectiveness in the collection and transportation process. To obtain a DBS sample, a simple finger prick is all that is needed; it does not require the presence of a trained phlebotomist for blood collection. This convenience extends to the possibility of collecting DBS samples outside clinical settings, reducing the burden on patients who would otherwise have to visit a clinic.

Following collection, DBS cards are left to air-dry overnight, then packaged and sent to a testing facility for analysis at room temperature. Unlike other methods, DBS specimens can be shipped to public health laboratories through standard mail, courier services, or express delivery as exempted specimens under the regulations issued by the International Air Transporter Association (IATA), the World Health Organization, and the International Civil Aviation Organization. DBS eliminates the need for expensive cold-chain transport and cryopreservation, as detailed in the CDC guidelines [25].

This streamlined approach significantly simplifies the pre-analytical phase of sample collection. Moreover, because DBS samples don’t require a cold-chain system to maintain their integrity, the overall cost of viral load testing in research studies is substantially reduced, making it a highly cost-effective option.

Performance assessment and implementation of DBS specimens for HIV-1 viral load testing have been the subject of numerous studies. The journey began with the WHO 2013 consolidated guidelines on antiretroviral drug use, which emphasized the need for higher detection thresholds when using DBS specimens for viral load testing, given uncertainties about their accuracy, especially below 1000 copies/mL.

In 2016, a study on DBS for HIV-1 viral load assays reported approximately 80% sensitivity and 90% specificity [26]. Misclassification rates were slightly lower in high viral load cases but ranged from 20.3% to 23.6% in low viral load cases. A 2017 study comparing DBS-fingerprint and DBS-venous methods found a sensitivity of 93% and a specificity of 95% for both DBS approaches [27]. In another 2017 study using Abbott and Roche platforms, DBS showed sensitivity and specificity of 93.9% and 88.0%, respectively, for Abbott platforms. Sensitivity was 100% for CAP/CTM, albeit with slightly lower specificity compared to Abbott [28]. A 2017 field evaluation in Kenya, a resource-limited setting, reported DBS performance at a threshold of ≥1000 copies/mL, with sensitivity ≥88.1% and specificity ≥93.1% [29]. A WHO pre-qualification study in 2018 reported DBS sensitivity of 76.0% and specificity of 89.7% at the 1000 RNA copies/mL threshold [21,30]. A cross sectional study comparing DBS HIV VL with plasma values on the Hologic Panther platform demonstrated a positive correlation of 0.96 [23]. A meta-analysis by the WHO and CDC in 2022 indicated that four out of six technologies using DBS samples for viral load testing achieved sensitivity and specificity above 83% at a treatment failure threshold of 1000 copies/mL. Notably, Abbott Real Time HIV-1, BioMerieux NucliSENS HIV-1, Roche COBAS TaqMan with FVE protocol, and Siemens VERSANT HIV-1 RNA performed exceptionally well under this threshold [11]. Furthermore, studies have highlighted the potential for enhanced DBS performance through the adoption of proper extraction protocols [24,31,32,33,34]. In summary, ongoing technological advancements are continually improving the reliability of DBS specimens in HIV-1 viral load testing (Table 2).

## 7. Feasibility Study Results of Implementation of DBS for Viral Load Testing in Different Setups

The feasibility of implementing the DBS specimen for expanding viral load programs has been rigorously examined by scientists worldwide, and the results suggest promising potential for its practical application in the field. Numerous studies conducted across various platforms have consistently highlighted the complementary nature of DBS as a valuable adjunct to plasma samples for improving access to viral load monitoring [11,12]. This complementarity is especially pronounced in resource-limited rural settings, where the advantages of DBS, such as its economical transport mechanism and ease of handling, become particularly significant. While DBS stands out as a crucial complementary tool, it is essential to recognize that, in resource-limited settings, it is often suggested as a primary approach due to its cost-effectiveness, simplified logistics, and potential to address challenges associated with traditional plasma-based methods. In India, where the HIV epidemic is primarily concentrated among high-risk groups and key populations like female sex workers, men who have sex with men, transgender individuals, and injecting drug users, the NACO initiated a study in 2012. This study identified the essential need for implementing DBS as an alternative sample type in India, taking into account its suitability for HIV-1 viral load testing [32]. DBS has been validated for HIV-1 viral load and HIV-1 DBS qualitative assays on various platforms, including the Indian Council of Medical Research-National AIDS Research Institute (ICMR-NARI) in India. A recent review published in 2022 by ICMR summarized the usage of dried blood specimens for screening sexually transmitted infections (STIs) and emphasized its role in expanding the HIV-1 viral load program to achieve the global 95-95-95 target in India [35]. In our proposed study, the research team has devised a protocol to assess the feasibility of alternative methods for viral load expansion in India, with a specific focus on the implementation capabilities of DBS in HIV-1 viral load expansion in the country [36]. A feasibility study conducted in Thailand in August 2021 reported a correlation coefficient of 0.62 between HIV viral load measured from plasma and DBS, with a mean difference of 0.02 (SD: 1.06) log10 IU/mL. At the threshold defining treatment failure (1000 copies/mL), the sensitivity of DBS HIV-1 RNA detection was 86% (95% CI, 74–94%). This study also demonstrated the high stability of HIV RNA in DBS after 2 to 4 days of shipping, compared to HIV RNA before shipping, even under varying temperature and humidity conditions [24]. A Vietnam feasibility study on dried blood spots for routine viral load monitoring, particularly to reach hard-to-reach populations, showed comparable results with plasma during virological failure and highlighted the usefulness of DBS in monitoring hard-to-reach patients during HIV care [13]. 

The feasibility of DBS for viral load testing has also been explored in the United States, where at-home DBS collection and longitudinal VL monitoring among low-ART-adherence MSM living with HIV showed promise. This mode of viral testing improved viral suppression in this group by simplifying sample collection and transport procedures for viral load testing [37]. Additionally, an African study reviewing progress in viral load expansion identified limitations related to transport conditions and turnaround times for specimen transfer to VL testing facilities. It discussed the need for implementing DBS and point-of-care (POC) testing, where applicable [24].

In a study conducted across seven PEPFAR-supported countries (Côte d’Ivoire, Kenya, Malawi, Mozambique, Namibia, South Africa, and Uganda) with a high pediatric HIV burden, an increasing proportion of VL tests were observed using dried blood spots, and lower sample rejection rates were noted for DBS compared to plasma [38]. A recent article published in AIDS Patient Care and STDs in 2023 asserted that DBS is a suitable and cost-effective tool for viral load monitoring when an ideal implementation protocol is followed to increase viral load testing in the population [39].

## 8. Principle of DBS-Based HIV-1 Viral Load Testing

**Figure 3:**
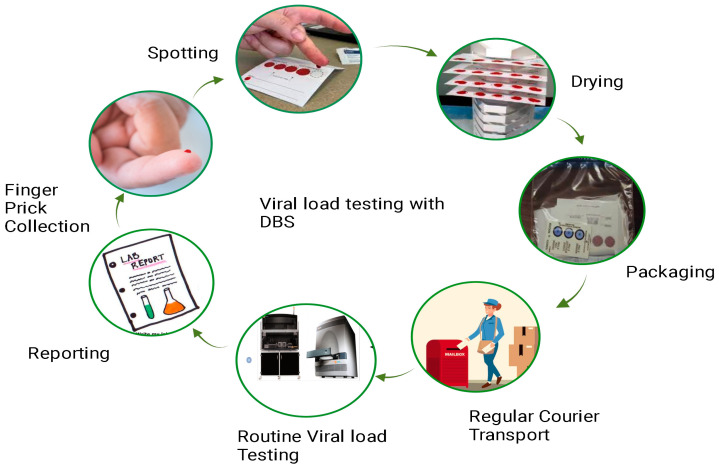
Workflow of dried blood spot viral load testing: This figure presents a detailed workflow illustrating the process of conducting viral load testing using DBS samples. The workflow begins with the collection of a small blood sample, typically obtained through a fingerstick. The DBS cards, on which the blood is applied, are then allowed to dry thoroughly. Subsequently, the dried blood spot cards are packaged and transported to the testing facility. Within the laboratory, the DBS samples undergo extraction of RNA and are subjected to molecular assays, such as polymerase chain reaction (PCR), to quantify HIV-1 RNA levels. The final steps involve result interpretation, contributing to a comprehensive understanding of the DBS viral load testing procedure.

The principle of DBS-based HIV-1 viral load testing revolves around the collection, drying, and subsequent analysis of a small volume of blood from an individual living with HIV (Figure 3). This method eliminates the need for immediate sample processing and enables easy transportation and storage.

### Methodology for Working with DBS

Dried blood spots are specimens prepared by collecting the blood spots from a finger or heel prick and spotting them directly onto a filter paper, which is then used for testing in the laboratory as a sample. The detailed process involves the following steps:

- **Blood Collection:** A small volume of blood, usually obtained through a finger prick, is collected onto a filter paper card specifically designed for DBS collection. The filter paper absorbs the blood and preserves it.
- **Drying:** The blood-soaked filter paper is allowed to air-dry completely, usually at room temperature. This step stabilizes the blood components on the filter paper, preventing degradation of the biological material.
- **Sample transport and storage:** Once dried, the DBS card can be stored at room temperature for an extended period. This eliminates the need for immediate cold-chain transportation and storage, making it particularly advantageous in resource-limited or remote settings.
- **Sample extraction and analysis:** To perform HIV-1 viral load testing, a small disc or punch is cut from the dried blood spot on the filter paper. This disc is then extracted to obtain the genetic material (RNA) of the virus. The extracted RNA is then subjected to nucleic acid amplification techniques, such as reverse transcription polymerase chain reaction (RT-PCR), to quantify the amount of HIV-1 viral RNA present in the blood.

The viral load determined through DBS analysis directly reflects the quantity of the virus present in an individual’s bloodstream. This metric holds immense significance as it serves as a critical gauge for assessing HIV progression and gauging the effectiveness of antiretroviral therapy. HIV-1 viral load testing through DBS analysis presents a streamlined, cost-efficient, and pragmatic means of monitoring the HIV-1 viral load, especially in regions grappling with limited resources or intricate logistical challenges.

## 9. Limitations of Working with DBS Specimen

Dried blood spot specimens, used for HIV-1 viral load PCR, have certain drawbacks. DBS utilizes whole blood as an input sample, resulting in the detection of pro-viral DNA and cell-associated RNA in addition to free HIV-1 RNA by RT PCR [40]. This sometimes leads to over-quantification. In response to this issue, the WHO recommended a higher threshold (3000–5000 copies/mL) for identifying treatment failure with DBS assays [41,42,43].

Furthermore, the smaller specimen volume used for DBS collection results in a lower limit of detection of 839 copies/mL [12]. This limitation can make it challenging to categorize patients for appropriate treatment. The inadequate lower limit of detection and the presence of pro-viral DNA are significant concerns with DBS specimens in the context of scaling up viral load testing. They introduce the risk of misclassification in cases of treatment failure and inconsistencies in results compared to the older standard of plasma specimens. 

Although the differences in dried blood spot nucleic acid extraction methods and viral load test chemistry employed by various platforms contribute to performance variability between test platforms, for instance, when compared with paired plasma specimen testing, few protocols like the FVE protocol associated with the standard Roche COBAS TaqMan SPEX exhibited significantly higher levels of specificity [44].

## 10. Conclusions and Future Directions

The data synthesized in this comprehensive review regarding the utilization of dried blood spots for HIV-1 viral load monitoring underscores the pivotal role of achieving virological suppression to combat HIV in India. This goal necessitates reaching viral load testing and treatment targets across all demographics, including marginalized populations and geographically challenging regions. Achieving these objectives calls for a concerted effort to address individual, societal, and structural barriers that hinder certain populations from accessing essential services. The present landscape reveals a concerning gap in viral load coverage due to limited access to testing facilities, particularly among vulnerable groups such as women, children, sex workers, and other people living with HIV in developing countries who often lack access to testing. The implementation of DBS analysis emerges as a potent tool for HIV-1 viral load monitoring, bridging this gap to reach those previously unreached. DBS has the potential to empower healthcare facilities by enhancing the accessibility of laboratory-based diagnostic tests. One major challenge in remote areas is the cost associated with sample transportation and cold-chain maintenance. Overcoming these hurdles demands the strategic implementation of policies for viral load testing, whether through DBS, plasma, or point-of-care methods when applicable. While DBS offers advantages in terms of cost, time, ease of sample collection, and transport, plasma retains its status as the standard sample type due to its lower detection limit and specificity at low viral load levels.

The systematic review of the available data underscores the necessity of complementary viral load monitoring methods in resource-limited settings. Striking a balance between utilizing plasma viral load testing in established antiretroviral treatment centers (ARTCs) and implementing DBS where testing facilities are lacking or transportation poses challenges is imperative. Beyond considerations of detection limits and proviral DNA, factors such as access to VL testing facilities, treatment implementation strategies, clinical relevance, the potential for low viremia in patients, clinical correlation with required VL monitoring, and the suitability of sample collection methods must be carefully evaluated. Furthermore, this review highlights the utility of point-of-care equipment for viral load testing, which, in conjunction with DBS and plasma, can be deployed as needed to maximize access to viral load testing for those in need.

In conclusion, while plasma will continue to serve as the gold standard for HIV-1 viral load testing in situations where feasibility and transportation are not obstacles, DBS emerges as a well-established alternative specimen in challenging and resource-limited regions for viral load testing. Striking the right balance between the convenience of reaching a broader population for HIV-RNA monitoring with a DBS-based assay and the risk of missing a few patients with low-level recurrent viremia is the key to effective HIV management in diverse settings. Key advancements in the field include the development of sensitive and specific assays tailored for DBS samples, addressing the challenges posed by limited sample volumes and potential inhibitory substances present in blood. Additionally, standardization efforts have been instrumental in ensuring consistency and reliability across different testing platforms and laboratories. However, considerations such as proper training, quality control measures, and appropriate result interpretation are essential to harnessing the full potential of this technology. In conclusion, DBS-based HIV-1 viral load testing has emerged as a transformative approach, offering a practical and accurate means of monitoring HIV-infected individuals. With ongoing research and technological refinements, DBS has the potential to play a pivotal role in scaling up HIV care and advancing towards global HIV elimination goals. Hence, this review underscores the importance of tailored approaches and continued research to advance HIV care and control in India and beyond.

## Figures and Tables

**Table 1:** Comparison of different modes and aspects of HIV-1 viral load testing.

Procedure	Plasma VL	DBS VL	CBNAAT POC	PSC VL
Sample collection: Requirement of phlebotomist and expensive consumables	Yes	No	Yes	Yes
Sample processing: Plasma separation using a centrifuge	No	No	Yes	No
Requirement of cold-chain transportation	Yes	No	No ^1^	No
Batch testing	Yes	Yes	Small batch	Yes
Proviral DNA contamination	No	Yes	No	No
Performance at a low viral load level	Good	Less	Good	Less
Restricted sample transport under the biohazard category	Yes	No	Yes	No
Time required for the test (per batch)	7–8 h	7–8 h	2 h ^2^	7–8 h

^1^ If the platform is available at the site of collection. ^2^ For a small batch size.

**Table 2:** Reported performance of DBS for HIV-1 viral load monitoring.

Assay Platform	VL Threshold	Sample Size	Sensitivity	Specificity	References
Abbott RealTime HIV-1, BioMerieux, Roche, and Siemens VERSANT	1000	10,871	>83%	>83%	[11]
Roche CAP/CTM, Abbott RealTime HIV-1, and the bioMérieux NucliSENS EasyQ	1000	323	80%	90%	[26]
Abbott RealTime HIV-1	1000	497	93%	95%	[27]
Abbott RealTime HIV-1	1000	200	93%	88%	[28]
Abbott RealTime HIV-1	1000	793	88.1%	93.1%	[29]
Abbott RealTime HIV-1	1000	323	76.0%	89.7%	[30]
Abbott RealTime HIV-1	2000	100	95%	Not reported	[31]
Abbott RealTime HIV-1	1000	130	50–100%	100%	[32]
Abbott RealTime HIV-1	1000	203	90.1%	96.2%	[33]
Abbott RealTime HIV-1	1000	87	94.8%	Not reported	[34]

## Data Availability

No new data were created during this study.
